# Risk factors for death and temporal trends in overall survival in patients with AIDS-associated primary central nervous system lymphoma (AIDS-PCNSL)

**DOI:** 10.1186/1750-9378-7-S1-O18

**Published:** 2012-04-19

**Authors:** Thomas S Uldrick, Sharon Pipkin, Susan Scheer, Nancy A Hessol

**Affiliations:** 1 HIV & AIDS Malignancy Branch, Center for Cancer Research, National Cancer Institute, National Institutes of Health, Bethesda, MD, USA; 2 San Francisco Department of Public Health, San Francisco, CA, USA; 3 Departments of Clinical Pharmacy & Medicine, University of California, San Francisco, CA, USA

## Background

AIDS-PCNSL is a rare EBV-associated B-cell neoplasm that continues to carry a poor prognosis, even in the highly active antiretroviral therapy (HAART) era. We hypothesized that overall survival (OS) is affected by prior diagnosis of central nervous system (CNS) infections as well as treatment approaches to both HIV and AIDS-PCNSL. We evaluated risk factors and temporal trends for OS in patients with AIDS-PCNSL.

## Methods

Adults with AIDS-PCNSL were identified through a computer linkage that matched AIDS case diagnosed between 1990-2000 from the San Francisco adult AIDS case registry with the California Cancer Registry (1985-2002), with mortality follow-up through 12/31/2007. Patients with non-B-cell histology or history of systemic non-Hodkgin lymphoma diagnosed within 2 years prior to AIDS-PCNSL diagnosis were excluded. Prognostic factors evaluated include: diagnosis of CNS infection prior to AIDS-PCNSL, diagnosis of other common opportunistic infection (OI) prior to AIDS-PCNSL (pneumocystis pneumonia [PCP] or mycobacterium avium complex [MAI]), pathologic versus clinical diagnosis, receipt of cancer therapy, HAART prescribed prior to or within 30 days of AIDS-PCNSL diagnosis, and year of diagnosis (1990-1995, 1996-1998, 1999-2002). Survival analyses employed Kaplan-Meier methodology.

## Results

**Figure 1:**
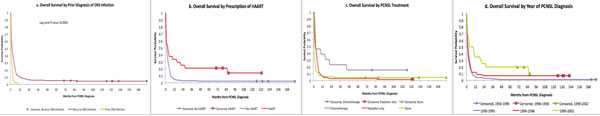


A total of 207 patients were identified, 96% male and 4% female. Median age 39 (IQR 35-46), 68% white, 21% black, 20% Hispanic, 2% Asian. Median CD4 20 cells/uL (IQR 6-53). HIV risk group: 79% MSM, 8% IDU, 9% MSM/IDU. CNS infections prior to AIDS-PCNSL: toxoplasmosis 8%, cryptococcus 9%, histoplasmosis 1%, extrapulmonary tuberculosis 1%. Treatment category: none 42%, radiation only 52%, chemotherapy 6% (5/13 chemotherapy only, 6/13 chemotherapy and radiation, 2/13 chemotherapy and immunotherapy). Risk factors for OS included prior CNS infection (p<0.0001), HAART (p=0.0023), AIDS-PCNSL treatment (p<0.0001), and calendar period of AIDS-PCNSL diagnosis (0.001), but not prior PCP or MAC (p= 0.23). (Figures 1 A-D.) OS was improved by HAART across treatment groups (p<0.0001).

## Conclusions

Prior diagnosis of CNS infection, HAART, and cancer treatment are strong predictors of OS. OS improved over time in these patients. Earlier diagnosis of AIDS-PCNSL and/or CNS infection, treatment of CNS infections, and cancer treatment that includes HAART and concomitant chemotherapy may increase AIDS-PCNSL survival. Prospective evaluation of curative-intent chemotherapy-based approaches to AIDS-PCNSL is urgently needed. Additional analyses are ongoing.

